# DNA methylation clock DNAmFitAge shows regular exercise is associated with slower aging and systemic adaptation

**DOI:** 10.1007/s11357-023-00826-1

**Published:** 2023-05-20

**Authors:** Matyas Jokai, Ferenc Torma, Kristen M. McGreevy, Erika Koltai, Zoltan Bori, Gergely Babszki, Peter Bakonyi, Zoltan Gombos, Bernadett Gyorgy, Dora Aczel, Laszlo Toth, Peter Osvath, Marcell Fridvalszky, Timea Teglas, Aniko Posa, Sylwester Kujach, Robert Olek, Takuji Kawamura, Yasuhiro Seki, Katsuhiko Suzuki, Kumpei Tanisawa, Sataro Goto, Csaba Kerepesi, Istvan Boldogh, Xueqing Ba, Kelvin J. A. Davies, Steve Horvath, Zsolt Radak

**Affiliations:** 1 Research Institute of Sport Science, Hungarian University of Sport Science, Budapest, Hungary; 2 Sports Neuroscience Division, Advanced Research Initiative for Human High Performance (ARIHHP), Faculty of Health and Sport Sciences, University of Tsukuba, Ibaraki, Tsukuba, 305-8574 Japan; 3 Laboratory of Exercise Biochemistry and Neuroendocrinology, Faculty of Health and Sport Sciences, University of Tsukuba, Ibaraki, Tsukuba, 305-8574 Japan; 4 Department of Biostatistics, Fielding School of Public Health, University of California Los Angeles, Los Angeles, CA 90095 USA; 5 Interdisciplinary Excellence Center, Department of Physiology, Anatomy and Neuroscience, Faculty of Science and Informatics, University of Szeged, 6700 Szeged, Hungary; 6 Department of Physiology, Faculty of Physical Education, Gdansk University of Physical Education and Sport, Gdańsk, Poland; 7 Department of Athletics, Strength and Conditioning, Poznań University of Physical Education, Poznań, Poland; 8 Faculty of Sport Sciences, Waseda University, Tokorozawa, 2-579-15 Japan; 9 Institute for Computer Science and Control (SZTAKI), Eötvös Loránd Research Network, Budapest, Hungary; 10 Department of Microbiology and Immunology, University of Texas Medical Branch at Galveston, Galveston, TX 77555 USA; 11 Key Laboratory of Molecular Epigenetics of Ministry of Education, Northeast Normal University, Changchun, Jilin, China; 12 Ethel Percy Andrus Gerontology Centre of the Leonard Davis School of Gerontology; Division of Molecular & Computational Biology, Department of Biological Sciences, of the Dornsife College of Letters, Arts, and Sciences; and Department of Biochemistry & Molecular Medicine of the USC Keck School of Medicine, University of Southern California, Los Angeles, CA 90089-0191 USA; 13 Department of Human Genetics, David Geffen School of Medicine, University of California Los Angeles, Los Angeles, CA 90095 USA; 14 Altos Labs, Cambridge Institute of Science, Cambridge, UK

**Keywords:** DNA methylation, DNAmFitAge, Regular exercise, Slower aging, Systemic adaptation

## Abstract

DNAmPhenoAge, DNAmGrimAge, and the newly developed DNAmFitAge are DNA methylation (DNAm)-based biomarkers that reflect the individual aging process. Here, we examine the relationship between physical fitness and DNAm-based biomarkers in adults aged 33–88 with a wide range of physical fitness (including athletes with long-term training history). Higher levels of VO_2_max (*ρ* = 0.2, *p* = 6.4E − 4, *r* = 0.19, *p* = 1.2E − 3), Jumpmax (*p* = 0.11, *p* = 5.5E − 2, *r* = 0.13, *p* = 2.8E − 2), Gripmax (*ρ* = 0.17, *p* = 3.5E − 3, *r* = 0.16, *p* = 5.6E − 3), and HDL levels (*ρ* = 0.18, *p* = 1.95E − 3, *r* = 0.19, *p* = 1.1E − 3) are associated with better verbal short-term memory. In addition, verbal short-term memory is associated with decelerated aging assessed with the new DNAm biomarker FitAgeAcceleration (*ρ*: − 0.18, *p* = 0.0017). DNAmFitAge can distinguish high-fitness individuals from low/medium-fitness individuals better than existing DNAm biomarkers and estimates a younger biological age in the high-fit males and females (1.5 and 2.0 years younger, respectively). Our research shows that regular physical exercise contributes to observable physiological and methylation differences which are beneficial to the aging process. DNAmFitAge has now emerged as a new biological marker of quality of life.

## Introduction

Aging is a natural process that depends on genetics, environment, and lifestyle factors. These factors make aging individualistic, and it has gained popularity in personalized medicine — especially through the use of epigenetic biomarkers. These biomarkers use a person’s DNA methylation to provide estimates of chronological age, biological age, rate of aging, mortality risk, etc. For example, DNAmPhenoAge and DNAmGrimAge are strong predictors of all-cause mortality and are associated with age-related diseases [1, 2]. Furthermore, DNA methylation biomarkers have been able to capture environmental effects on aging. Monozygotic twins with different aging diseases have a different DNAmAge Acceleration [3, 4], which underlines the significant role environmental and lifestyle factors have on aging phenotypes.

One of the striking effects of aging is a decrease in many physiological functions, and it has been shown that age-associated decline can be attenuated with regular physical activity [5]. Regular exercise also decreases mortality risk and the incidence of age-related diseases including dementia, Alzheimer’s disease, osteoporosis, hypertension, cardiovascular diseases, cancer, stroke, and arthritis [6]. Moreover, regular physical activity has systemic effects on the body, influencing almost all organ functions, cellular and organ metabolism, redox-sensitive cellular signaling, and activation of the immune system [6].

This systemic adaptation provides a rationale for DNA how methylation can be influenced by physical exercise, and for physical activity to be a valuable component of DNAm-based aging biomarkers. However, only recently has physical activity been incorporated into a DNAm-based aging biomarker. DNAmFitAge, a new DNAm biomarker, provides an estimate of biological age using DNAm-based estimates of three physical fitness measurements: maximal oxygen uptake (DNAmVO_2_max), maximal gripping force (DNAmGripmax), and gait speed (DNAmGaitspeed) [7]. While DNAmFitAge was validated in healthy adult populations, it was unknown how this new biomarker would perform in trained and physically fit individuals, or if it would capture epigenetic differences related to physical fitness.

Here we investigated the complex relationship among DNAm-based biomarkers of aging, including DNAmFitAge, a variety of physiological functioning variables, and blood serum measures including cholesterol, irisin level, and redox balance on 303 healthy individuals aged between 33 and 88 years with diverse levels of physical fitness. Our research indicates that regular physical exercise-related adaptation is related to methylation differences which are both beneficial to aging and closely related to physiological functions.

## Methods

### Study population

The study was approved by the National Public Health Center in accordance with the Helsinki Declaration and the regulations applicable in Hungary (25167-6/2019/EÜIG). The subjects of this study were volunteers who signed a written consent form to participate in the investigation. A large number of volunteers (*n* = 205) participated in the World Rowing Masters Regatta in Velence, Hungary. In all, a total of 303 subjects, between the ages of 33 and 88, were included in the study. Subjects completed a questionnaire regarding their health, educational status, and lifestyle, including exercise habits. The master rowing group was very heterogeneous; many athletes had just one or two training sessions a week, while others had daily training. Therefore, we classified subjects into different fitness groupings based on the level of VO_2_max, which represents cardiovascular fitness. VO_2_max has been regarded as one of the best indicators of an athlete’s physical capacity. Hence, subjects were divided into one of two fitness categories: medium-low-fit group (MED-LOW FIT) (male *n* = 50, female *n* = 62) or highly fit (HIGH-FIT) group (male *n* = 93 and female *n* = 91) based on the 75th percentile of the VO_2_max values (Table 1).

**Table 1:** Characteristics of subjects and mean values of measured parameters

		Female med-low	Female high	Male med-low	Male high
Age	*n*	63	91	52	97
	Mean	64.651	58.132	60.173	60.608
	SD	11.929	8.597	13.279	11.210
VO_2_max (Est.)	*n*	50	91	47	97
	Mean	28.101	42.542	35.051	50.154
	SD	5.435	7.520	6.060	10.029
MaxGrip (kg)	*n*	63	91	52	97
	Mean	28.240	31.701	49.473	50.545
	SD	6.024	5.567	11.177	8.456
MaxJump (cm)	*n*	62	91	50	97
	Mean	22.061	26.022	30.316	33.427
	SD	6.687	5.737	8.259	7.238
BMI	*n*	63	91	51	97
	Mean	26.526	24.340	27.312	25.474
	SD	4.968	3.436	3.678	2.556
VSTM	*n*	62	91	52	96
	Mean	5.806	6.176	5.942	6.458
	SD	1.304	1.226	1.406	1.305
FitAge	*n*	62	91	50	93
	Mean	69.599	61.639	63.172	61.949
	SD	12.481	9.042	13.232	10.919
FitAgeAccel	*n*	62	91	50	93
	Mean	1.783	0.241	0.263	− 1.600
	SD	3.631	3.094	2.910	3.487
GrimAge	*n*	62	91	50	93
	Mean	62.768	57.136	60.753	61.107
	SD	9.799	7.236	11.026	9.103
GrimAgeAccel	*n*	62	91	50	93
	Mean	− 0.696	− 1.186	1.170	1.067
	SD	2.947	2.952	2.822	2.718
PhenoAge	*n*	62	91	50	93
	Mean	52.850	46.857	48.306	48.827
	SD	12.375	8.274	13.145	10.720
PhenoAgeAccel	*n*	62	91	50	93
	Mean	0.206	− 0.097	0.011	− 0.048
	SD	5.739	5.150	5.032	4.809
LDL	*n*	63	91	50	94
	Mean	3.797	3.546	3.677	3.405
	SD	0.961	0.858	0.772	0.752
HDL	*n*	63	91	50	94
	Mean	1.634	1.877	1.373	1.654
	SD	0.394	0.387	0.281	0.336
Redox balance	*N*	59	69	41	64
	Mean	5.159	5.569	6.057	6.415
	SD	0.995	0.979	1.009	0.936
Irisin	*n*	29	57	25	44
	Mean	11.759	12.450	12.441	13.446
	SD	2.438	2.349	2.225	1.936

### Physiology tests

Digit span test was applied to assess the working memory, where larger values indicate better memory. Maximum hand gripping force is often used to measure of age-associated declines in general muscle strength. The dynamic strength of the legs was assessed by the maximum vertical jump, using a linear encoder. Body mass index was apprised by body composition monitor BF214 (Omron, Japan). Maximal oxygen uptake, VO_2_max, measures the volume of oxygen the body processes during incremental exercise in milliliters used in 1 min of exercise, per kilogram of body weight (mL/kg/min), and was estimated through Chester step test. Participants were classified into fitness groups by already established sex and age strata-specific VO_2_max reference standards [8]. Accordingly, subjects were divided into either a medium-low-fit group (MED-LOW FIT) male (*n* = 50), female (*n* = 62), or a high-fit (HIGH-FIT) group male (*n* = 93) and female (*n* = 91), based on the age- and gender-specific 75th percentile of the VO_2_max values.

### Determination of hematologic and biochemical variables

Blood samples were collected before the subjects performed the VO_2_max evaluation test, and were stored in evacuated tubes containing EDTA as an anticoagulant for determination of erythrogram. Blood samples were centrifuged and stored at − 80 °C.

### Measurement of irisin

Plasma irisin was quantified using commercially available ELISA kits (EK-067–29, Irisin Recombinant, Phoenix Pharmaceuticals, Inc, Burlingame, USA). All samples from a particular subject were analyzed using the same plate (intra-assay). Intra- and inter-assay coefficients of variation were 4.1% and 15.2%, respectively.

### Assessment of redox balance

The total amount of organic hydroperoxides in blood was spectrophotometrically estimated using the d-Roms (derivatives of reactive oxygen metabolites) test as described previously [9]. The concentrations are expressed in conventional units (Carratelli units; UCarr) in which 1 UCarr corresponds to 0.8 mg/L H_2_O_2_. The d-Roms test is performed using a FREE Carpe Diem analyzer (Wismerll Co., Ltd., Tokyo, Japan) [9]. The plasma ferric-reducing ability was estimated using the biological antioxidant power test (BAP). In brief, ferric chloride is mixed with a special chromogen substrate, a thiocyanate derivative. Plasma (10 μL) prepared from each blood sample is added to this reaction mixture and incubated at 37 °C for 5 min. The reduction of ferric ion is quantified by measuring the absorbance change at 505 nm. The BAP assays are also performed on a FREE Carpe Diem analyzer [9]. The redox balance was estimated by the BAP/dROM ratio.

### Measurement of DNA methylation

Epigenome-wide DNA methylation 85 K was measured with the Infinium MethylationEPIC BeadChip (Illumina Inc., San Diego, CA) according to the manufacturer’s protocol. In short, 500 ng of genomic DNA was bisulfite converted using the EZ-96 DNA Methylation MagPrep Kit (Zymo Research, Irvine, CA, USA) with the KingFisher Flex robot (Thermo Fisher Scientific, Breda, Netherlands). The samples were plated in randomized order. The bisulfite conversion was performed according to the manufacturer’s protocol with the following modifications: for binding of the DNA 15 µL MagBinding Beads was used. The conversion reagent incubation was done according to the following cycle protocol: 16 cycles of 95 °C for 30 s followed by 50 °C for 1 h. After the cycle protocol the DNA was incubated for 10 min at 4 °C. Next, DNA samples were hybridized on the Infinium MethylationEPIC BeadChip (Illumina Inc., San Diego, CA) according to the manufacturer’s protocol with the modification that 8 µL bisulfite-treated DNA was used as start material.

Quality control of the DNA methylation data was performed using minfi, Meffil, and ewastools packages with R version 4.0.0. Samples that failed technical controls, including extension, hybridization, and bisulfite conversion, according to the criteria set by Illumina, were excluded. Samples with a call rate < 96% or at least with 4% of undetected probes were also excluded. Probes with a detection *p* value > 0.01 in at least 10% of the samples were set as undetected. Probes with a bead number < 3 in at least 10% of the samples were excluded. We used the “noob” normalization method in R to quantify methylation level [10]. The details on the processing of DNAm data and the calculation of the measures of aging, or pace of aging, were calculated using Horvath’s online age calculator (https://dnamage.genetics.ucla.edu/).

### Epigenetic biomarkers

The development of epigenetic clocks is reviewed in Horvath and Raj [2]. Epigenetic clocks are considered highly promising molecular biomarkers of aging. The most commonly used epigenetic clocks are Hannum’s blood-specific clock [11] and Horvath’s pan-tissue clock [12], which are based on levels of DNAm at 71 and 353 CpG sites, respectively. These clocks are highly correlated with chronological age, and the discrepancy resulting from the regression of DNAm age on calendar age—referred to as epigenetic age acceleration—is associated with an increased risk of all-cause mortality. First-generation epigenetic clocks, such as the pan-tissue clock from Horvath 2013, exhibit statistically significant but relatively weak associations with clinical biomarkers and mortality risk. Far stronger associations with mortality risk and a host of age-related conditions can be observed with so-called second-generation epigenetic clocks such as PhenoAge [13] and GrimAge [14]. GrimAge has thus far appeared to be the most predictive model for mortality risk estimator [14]. The DunedinPACE clock was estimated as described by Belsky et al. [15].

DNAmFitAge represents a new epigenetic biomarker that incorporates physical fitness. The creation of DNAmFitAge was recently described [7]. In short, DNAm fitness biomarkers were created using LASSO penalized regression (DNAmGaitspeed, DNAmGripmax, DNAmFEV1, and DNAmVO_2_max). The DNAm fitness biomarkers for gait speed, gripmax, and FEV1 are built for either males or females and have two versions each. One version uses chronological age and DNA methylation to form an estimate of the fitness parameter, and the other version uses DNA methylation only to form estimates. In this paper we evaluated both the age and no-age versions. DNAmVO_2_max uses age as a covariate and is built for both sexes. DNAmFitAge combines four DNAm-based biomarker variables, including three of the DNAm fitness biomarkers: DNAmGripmax noAge, DNAmGaitSpeed noAge, and DNAmVO_2_max, and DNAmGrimAge, a biomarker of mortality risk [7]. Finally, FitAgeAcceleration is the age-adjusted estimate of DNAmFitAge formed from taking the residuals after regressing DNAmFitAge onto chronological age. FitAgeAcceleration provides an estimate of epigenetic age acceleration, i.e., how much older or younger a person’s estimated biological age is from the expected chronological age (Table 1).

### Statistical analysis

The relationships between target and predictor variables were evaluated using multiple linear regression controlling for age and sex. Analyses were conducted using Statistica 13 software (TIBCO). For the analysis of irisin, a variable for plate was included to control for possible batch effects. Fitness group differences were investigated by two-way ANOVA using sex and fitness group as factors; group means were compared by Tukey’s HSD. If data did not follow the normal distribution, assessed by Shapiro-Wilk test, the Kruskal-Wallis test was applied instead. The association between verbal short-term memory and biochemical/physiological markers was analyzed by calculating Spearman’s rho and Kendall’s tau.

### Physical fitness to DNAm biomarkers

We used two-sample *t*-tests and non-parametric Kruskal-Wallis tests to determine if DNAm biomarkers were significantly different between the high-fit and low-med-fit groups in males and females (Table 1). We use the age-adjusted DNAm variables (FitAge Acceleration, GrimAge Acceleration, and PhenoAge Acceleration) to remove any age effect seen between groups. The same *t*-tests and Kruskal-Wallis tests were performed for physical fitness parameters Gripmax and Jumpmax (relative and absolute) to provide a reference for the DNAm-based surrogates.

VO_2_max is excluded from the table because VO_2_max was used to form high-fit and low-med-fitness groups. Furthermore, DNAmVO_2_max is excluded from the table because subjects from the study were used to construct the DNAmVO_2_max biomarker so observed differences by group are simply artifacts of the training dataset.

## Results

### Age-related physiological functioning and blood markers

**Fig. 1:**
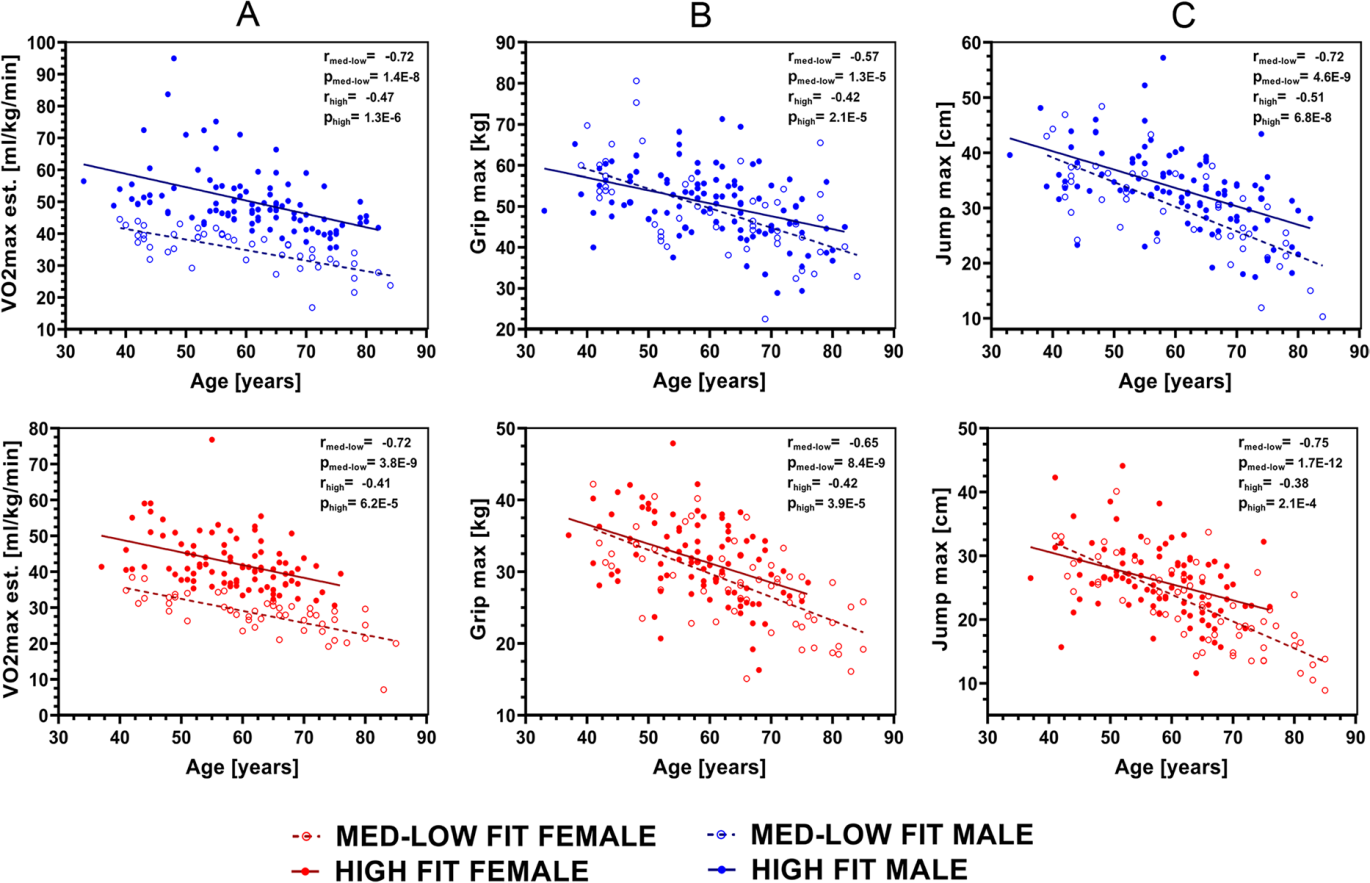
Age-related decline in VO_2_max (**A**), GripMax (**B**), and JumpMax (**C**) of male and female HIGH-FIT and MED-LOW-FIT subjects. Regardless of fitness level VO_2_max, GripMax, and JumpMax decreased as a result of aging; however, the decrease started from higher values and subjects from the HIGH-FIT group reached the values of MED/LOW-FIT subjects who were chronologically more than 20 years older. Pearson correlation coefficient was calculated for male and female subjects in fitness category as marked (for VO_2_ max: male HIGH-FIT *n* = 97, female HIGH-FIT *n* = 91, male MED-LOW-FIT *n* = 47, female MED-LOW *n* = 50; for Gripmax: male HIGH-FIT *n* = 97, female HIGH-FIT *n* = 91, male MED-LOW-FIT *n* = 52, female MED-LOW *n* = 63; for Jumpmax: male HIGH-FIT *n* = 97, female HIGH-FIT *n* = 91, male MED-LOW-FIT *n* = 50, female MED-LOW *n* = 62)

**Fig. 2:**
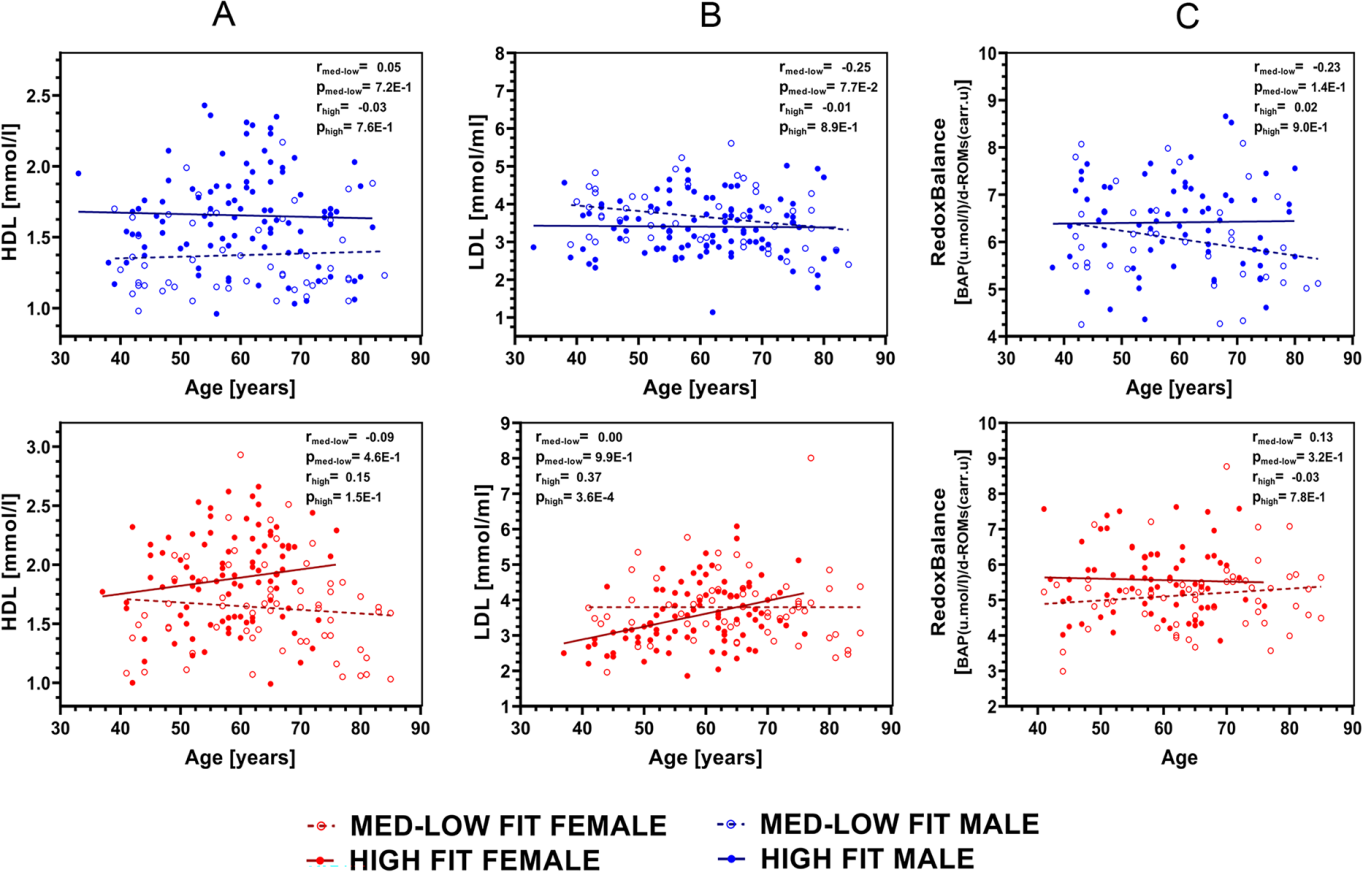
Age-associated changes in HDL (**A**), LDL (**B**), and redox balance (**C**) of male and female HIGH-FIT and MED/LOW-FIT subjects. In HIGH-FIT males HDL levels were higher than in MED/LOW-FIT group and age-associated changes were not significant. In females, both HDL and LDL levels tended to increase with aging. The redox balance remained unaltered by aging for both genders. Pearson correlation coefficient was calculated for male and female subjects in fitness category as marked (for HDL max: male HIGH-FIT *n* = 94, female HIGH-FIT *n* = 91, male MED-LOW-FIT *n* = 50, female MED-LOW *n* = 63; for LDL: male HIGH-FIT *n* = 94, female HIGH-FIT *n* = 91, male MED-LOW-FIT *n* = 50, female MED-LOW *n* = 63; for redox balance: male HIGH-FIT *n* = 64, female HIGH-FIT *n* = 69, male MED-LOW-FIT *n* = 41, female MED-LOW *n* = 59)

**Fig. 3:**
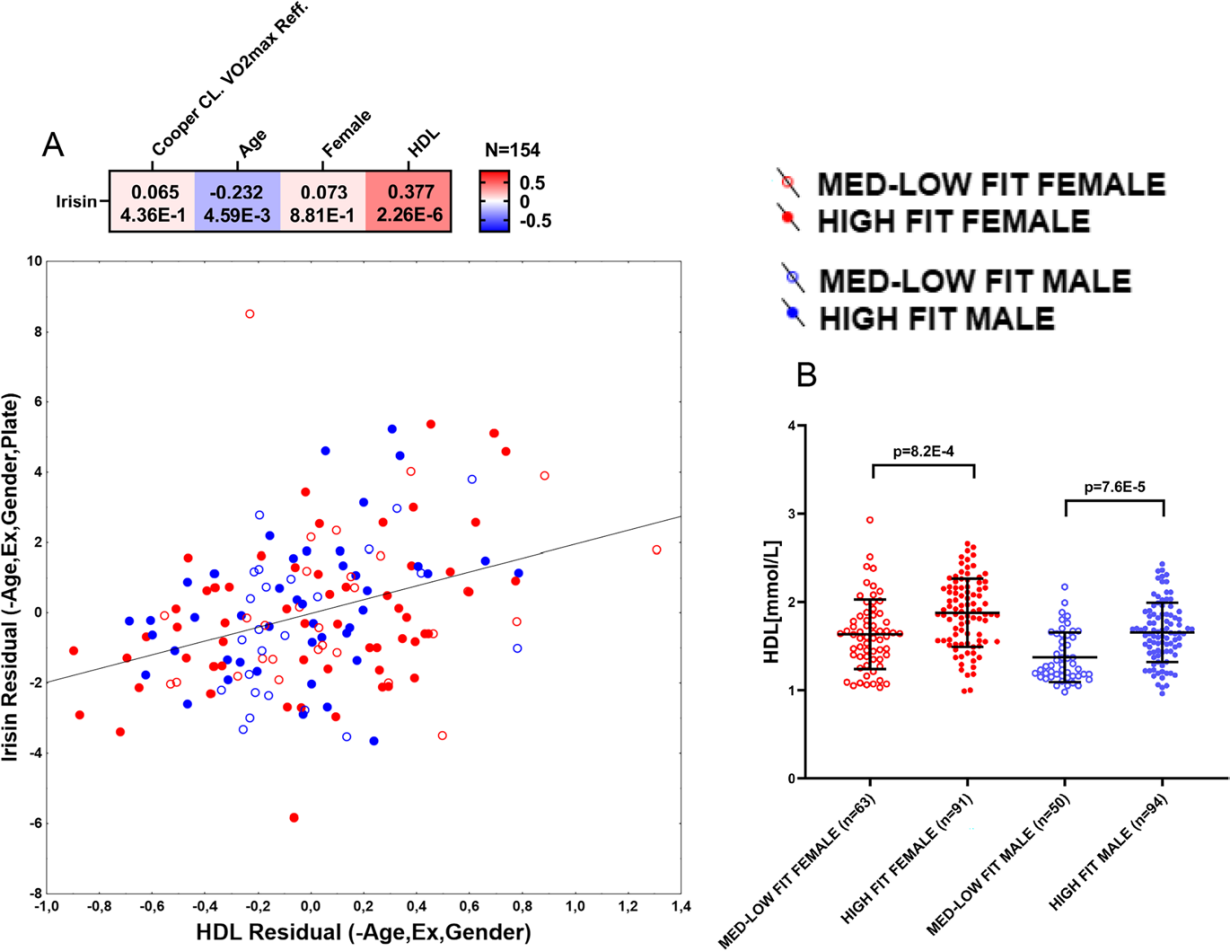
Correlation between HDL and irisin (**A**) and fitness- and gender-associated levels of HDL (**B**). Heatmap shows the correlation between irisin and HDL levels adjusted for age, gender, fitness level, and batch (data not shown) with corresponding partial *r* (r_p_) and *p* values. The Cartesian graph shows the residuals of irisin ~ age + gender + fitness level + plate model against residuals of HDL ~ age + gender + fitness level model. Grouped plots show the data distribution and group differences in HDL levels. Data were analyzed by multiple regression; group differences were calculated by Kruskal-Wallis test (male HIGH-FIT *n* = 44, female HIGH-FIT *n* = 57, male MED-LOW-FIT *n* = 24, female MED-LOW *n* = 29)

Aging resulted in a decline in all measured physiological functions in MED-LOW FIT and HIGH-FIT groups (Fig. 1). The rate of decline may be slower for high-fit groups, especially at older ages, but only the age-associated decline in Jumpmax differs by fitness level. Interestingly, Jumpmax, which is used to evaluate anaerobic power, is the only measured physiological function in which the decline is attenuated by fitness level, where high-fit individuals have a slower decline. The change in LDL, HDL, and redox balance by physical fitness and sex are shown in Fig. 2. HDL appears constant in males across age in either fitness group, and the high-fit males have consistently higher HDL levels than med-low fit males. The top panel of Fig. 3 presents the coefficients and *p* values from multiple linear regression for serum irisin levels; irisin levels decrease with age (0.23 average decrease for every 1 year older). Furthermore, Fig. 3 shows that HDL is positively associated with irisin and that HDL is significantly different between high-fit and low/med-fit males and females.

**Fig. 4:**
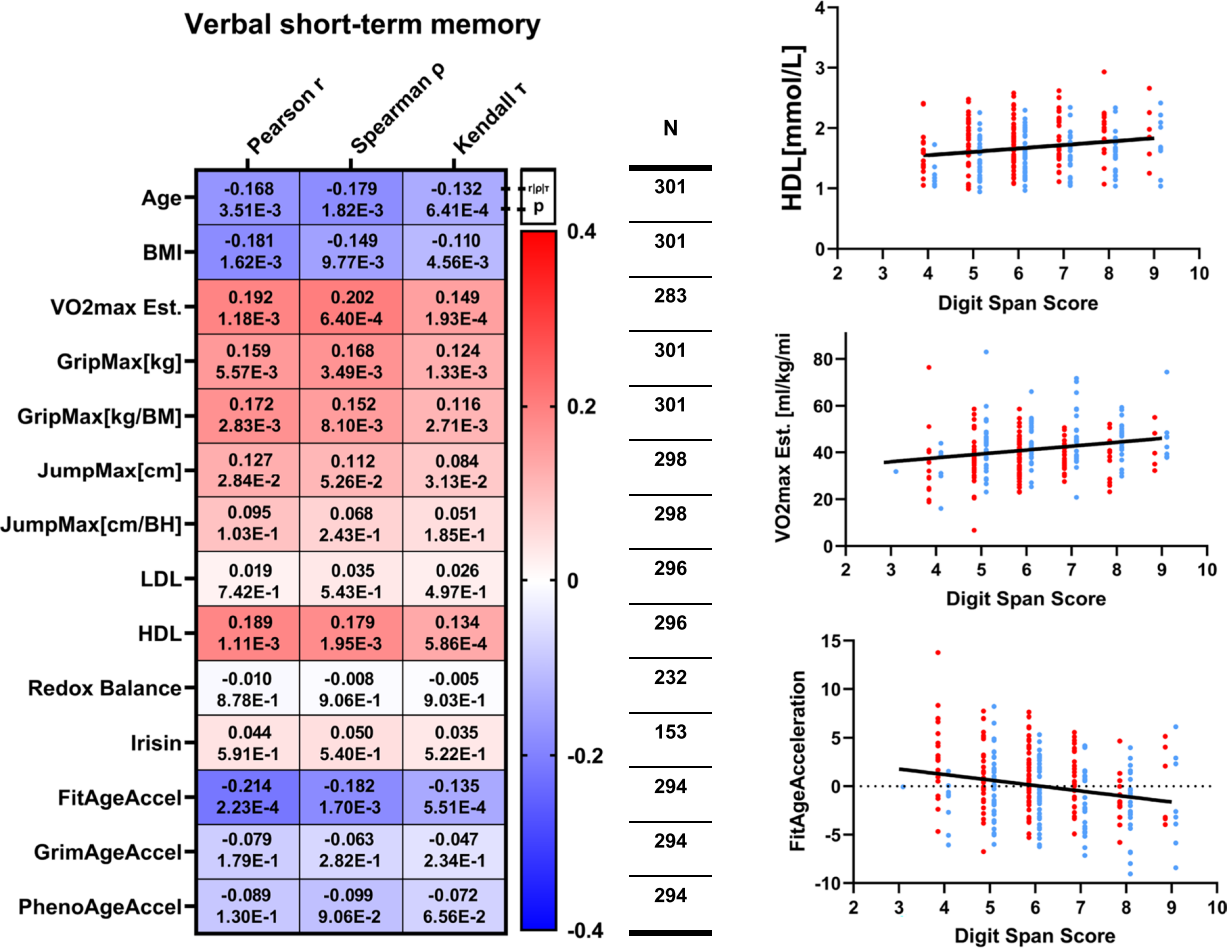
The association between verbal short-term memory and physiological, biochemical, and DNAm-based parameters. On the heatmap Pearson’s *r*, Spearman’s rho, and Kendal’s tau and corresponding *p* values are presented. The scatterplots show the digit snap scores in respect of marked physiological parameters, red dots mark the female subjects, and blue dots mark the male subjects (age, BMI, GripMax: male HIGH-FIT *n* = 96, female HIGH-FIT *n* = 91, male MED-LOW-FIT *n* = 52, female MED-LOW *n* = 62; VO_2_Max: male HIGH-FIT *n* = 96, female HIGH-FIT *n* = 91, male MED-LOW-FIT *n* = 47, female MED-LOW *n* = 49; JumpMax: male HIGH-FIT *n* = 96, female HIGH-FIT *n* = 91, male MED-LOW-FIT *n* = 50, female MED-LOW *n* = 61; LDL, HDL: male HIGH-FIT *n* = 93, female HIGH-FIT *n* = 91, male MED-LOW-FIT *n* = 50, female MED-LOW *n* = 62; DNAmAge Accelerations: male HIGH-FIT *n* = 92, female HIGH-FIT *n* = 91, male MED-LOW-FIT *n* = 50, female MED-LOW *n* = 61; redox balance: male HIGH-FIT *n* = 64, female HIGH-FIT *n* = 69, male MED-LOW-FIT *n* = 41, female MED-LOW *n* = 58; irisin: male HIGH-FIT *n* = 43, female HIGH-FIT *n* = 57, male MED-LOW-FIT *n* = 25, female MED-LOW *n* = 28)

The best short-term memory scores were observed in younger, leaner, and more physically fit individuals (Fig. 4). The digit span test is the only physiological test independent of sex, which provides a measurement of verbal short-term memory. Higher levels of VO_2_max (*p* = 0.0013), JumpMax (*p* = 0.028), GripMax (*p* = 0.0056), and HDL levels (*p* = 0.0011) were associated with better verbal short memory. Importantly, the newly created FitAgeAcceleration is also negatively correlated with digit span test results (*p* = 0.0002), meaning better verbal memory is associated with decelerated aging.

**Fig. 5:**
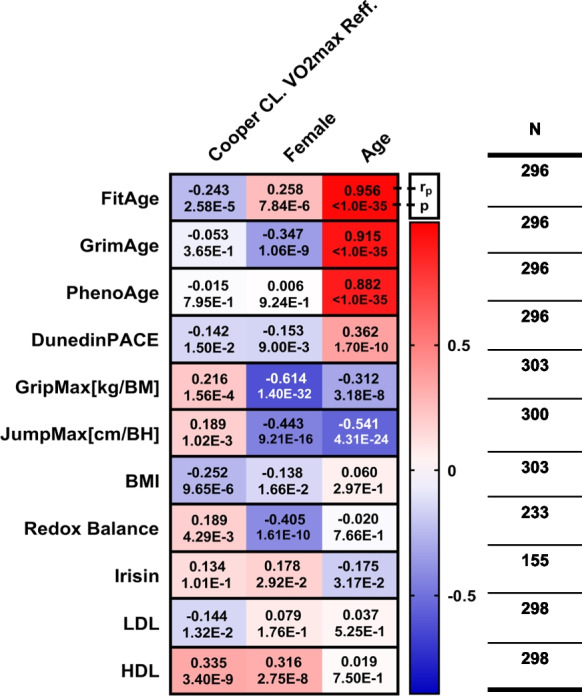
The relationship between measured and calculated parameters for fitness level, gender, and age. Results of the multiple linear regression models can be found in each cell. Row titles are the dependent variables and column titles are the independent variables in the model. Each row represents a multiple linear regression model and cells contain partial correlation coefficients (*r*_p_) and corresponding *p* values. The *n* values at the end of each row show the available sample size for all four variables in the model (DNAmAge biomarkers: male HIGH-FIT *n* = 93, female HIGH-FIT *n* = 91, male MED-LOW-FIT *n* = 50, female MED-LOW *n* = 62; GripMax, BMI: male HIGH-FIT *n* = 97, female HIGH-FIT *n* = 91, male MED-LOW-FIT *n* = 52, female MED-LOW *n* = 63; JumpMax: male HIGH-FIT *n* = 97, female HIGH-FIT *n* = 91, male MED-LOW-FIT *n* = 50, female MED-LOW *n* = 62; redox balance: male HIGH-FIT *n* = 64, female HIGH-FIT *n* = 69, male MED-LOW-FIT *n* = 41, female MED-LOW *n* = 59; LDL and LDL: male HIGH-FIT *n* = 94, female HIGH-FIT *n* = 91, male MED-LOW-FIT *n* = 50, female MED-LOW *n* = 63; irisin: male HIGH-FIT *n* = 44, female HIGH-FIT *n* = 57, male MED-LOW-FIT *n* = 25, female MED-LOW *n* = 29)

The relationships between physiological, biochemical, and DNAm biomarkers with age, sex, and physical fitness were as expected (Fig. 5). All of the DNAm-based biomarkers: DNAmFitAge, DNAmGrimAge, and DNAmPhenoAge, had strong relationships with chronological age (*p* < 1.0E − 35). However, the correlation between DunedinPACE and age and VO_2_max was less powerful than DNAmFitAge. The measured physiological tests: VO_2_max, JumpMax, and GripMax, had negative associations with age, and females had a lower mean value than did males. Importantly, high-fit individuals (classified through VO_2_max) were associated with younger DNAmFitAge (*p* = 2.6E − 5), lower BMI (*p* = 9.7E − 6), stronger relative GripMax (*p* = 1.6E − 4), farther JumpMax (*p* = 2.6E − 4), higher HDL levels (*p* = 3.4E − 9), and higher redox balance (*p* = 4.3E − 3).

### DNAmFitAge and other DNAm biomarkers

**Fig. 6:**
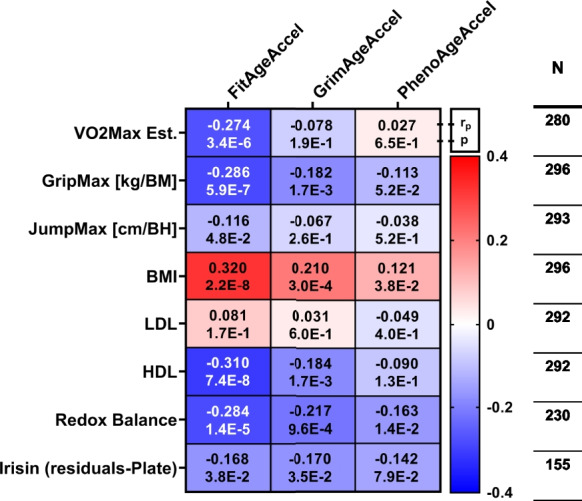
The relationship between physiological and biochemical parameters with acceleration of FitAge, GrimAge, and PhenoAge. Results of the multiple linear regression models can be found in each cell adjusted for gender. Each row (dependent variable) and column (independent variable) intersection represents a multiple linear regression model (with gender variable that is not shown) and cells contain partial correlation coefficients (*r*_p_) and corresponding *p* values. The *n* values at the end of each row show the available samples for all 3 in the model. For irisin, batch adjusted residual values were included for the multiple regression model (VO_2_max: male HIGH-FIT *n* = 93, female HIGH-FIT *n* = 91, male MED-LOW-FIT *n* = 46, female MED-LOW *n* = 50; GripMax, BMI: HIGH-FIT *n* = 93, female HIGH-FIT *n* = 91, male MED-LOW-FIT *n* = 50, female MED-LOW *n* = 62; JumpMax: male HIGH-FIT *n* = 93, female HIGH-FIT *n* = 91, male MED-LOW-FIT *n* = 48, female MED-LOW *n* = 61; redox balance: male HIGH-FIT *n* = 63, female HIGH-FIT *n* = 69, male MED-LOW-FIT *n* = 40, female MED-LOW *n* = 58; LDL and LDL: male HIGH-FIT *n* = 91, female HIGH-FIT *n* = 91, male MED-LOW-FIT *n* = 48, female MED-LOW *n* = 62; irisin: male HIGH-FIT *n* = 44, female HIGH-FIT *n* = 57, male MED-LOW-FIT *n* = 25, female MED-LOW *n* = 29)

FitAge Acceleration had the most powerful relationship to physical fitness parameters, BMI, and blood serum markers compared with GrimAge and PhenoAge Acceleration (Fig. 6). Moreover, the direction of effect was as expected. A positive FitAge Acceleration corresponded to an older estimated biological age than the true chronological age (+ = older), whereas a negative FitAge Acceleration indicated that a person was younger biologically than they were chronologically (− = younger/fitter). For every 1-year increase in FitAge Acceleration, there was an average 0.29 decrease in relative grip strength (kg force/body mass), 0.12-cm decrease in relative jumping distance (cm distance/body height), 0.32 increase in body mass index, 0.31 decrease in blood HDL, 0.28 decrease in redox balance, and 0.17 increase in blood irisin. The direction of effect was generally conserved across FitAge, GrimAge, and PhenoAge Acceleration, but the significance and magnitude of effects was stronger with FitAge Acceleration.

FitAge Acceleration, but not GrimAge or PhenoAge Acceleration, distinguished high-fit subjects from low/med-fit subjects in males and females (Table 1). FitAge Acceleration was 1.5 years younger on average in high-fit females compared to low/med-fit females (*p* = 0.005), and FitAgeAcceleration was 2.0 years younger on average in high-fit males compared to low/med-fit males (*p* = 0.0007). GrimAge Acceleration and PhenoAge Acceleration estimated younger values in the high-fit groups of males and females, but neither were statistically significantly different (0.25 < *p* < 0.82). Furthermore, the differences observed between female fitness groups with GrimAge and PhenoAge Acceleration had smaller magnitudes (0.5 and 0.2, respectively) than did FitAge Acceleration. Therefore, highly fit females and highly fit males were estimated to be 1.5 and 2.0 years biologically younger, on average, than their low- to medium-fit counterparts, suggesting that regular physical exercise is protective of biological age in both males and females.

### DNAm fitness biomarkers

The underlying DNAm fitness biomarkers DNAmGripmax, DNAmGaitSpeed, and DNAmFEV1 could distinguish high-fit females from low/med-fit females but did not distinguish fitness grouping in males. Regardless of whether or not chronological age was used in the DNAm fitness biomarker construction, each biomarker had similar estimated differences in female fitness groups: 1.49 and 1.44 for DNAmGripmax, 0.10 and 0.09 for DNAmGaitspeed, and 0.14 and 0.15 for DNAmFEV1. We hypothesize that the DNAm fitness biomarkers could not detect differences in the male fitness groups because the true physical fitness parameters were not very distinct between the male groups (relative gripmax *p* = 0.049, jumpmax *p* = 0.070).

## Discussion

Lifestyle choices including healthy nutrition and regular exercise reduce mortality risk and arguably slow the aging process. Here, we evaluated the new DNA methylation-based biomarker, DNAmFitAge, which relates physical exercise to the epigenome, in healthy and athletic adults. DNAmFitAge along with previously published epigenetic mortality risk estimators DNAmGrimAge and DNAmPhenoAge predict coronary heart disease risk, comorbidities, and disease-free status [7]. However, here we demonstrate that FitAge Acceleration is better able to capture both general and individual methylation-based alterations from exercise-induced adaptations. FitAge Acceleration has a stronger relationship with verbal memory, HDL, BMI, redox balance, VO_2_max, JumpMax, and GripMax. These findings support the hypothesis that the effect or improvement of DNAmFitAge would be more pronounced in athletes, and further validates the potential of this new biomarker that incorporates physical fitness through DNA methylation. The relationship between FitAge Acceleration and verbal memory support expansive research relating the benefits of exercise on cognitive function. Our results are the first to demonstrate that DNAmFitAge is not just related to athletic status, but that it can also be an indicator of cognitive health. This finding provides another potential utility area for this new biomarker.

The present investigation revealed that long-term regular exercise attenuates the age-associated decline in physiological function, including verbal short-term memory, overall body strength, explosive strength, and cardiovascular fitness. This may suggest that individuals with higher levels of physiological function could have a higher physiological quality of life and a decreased risk of mortality [16]. Furthermore, DNAmFitAge shows that higher levels of physiological functioning correspond to decelerated aging. FitAge Acceleration estimates that high-fit individuals have a 1.5 to 2.0 younger biological age on average compared to low/med-fit individuals in females and males, respectively. These findings further support the hypothesis that regular exercise is protective of health and beneficial to the aging process. Moreover, it must be mentioned that our low/medium-fitness group’s physical fitness level most probably were better than the average for the general population. The values of DNAmFitAge and DNAmFitAge acceleration remained to be tested in subjects with poor levels of physical fitness. In addition, further investigations will be required in order to explore the effects of childhood physical activity on the progress of DNAmFitAge.

HDL and irisin have complex roles in physiology, and the positive relationship we observe between physical exercise, HDL, and irisin align with the protective effects seen between HDL and irisin on glucose homeostasis. HDL is involved in neuroprotection, inflammation, oxidative stress, nitric oxide production, and regulation of plasma glucose homeostasis [17]. Infusing HDL into skeletal muscle can help control glucose uptake in people with type 2 diabetes mellitus [18]. Additionally, irisin helped protect against high glucose cytotoxicity and preserved crucial AMPK-insulin receptor signaling in cells treated with C2C12 [19]. This molecular interaction may potentially explain the protective effects of exercise on higher HDL and irisin on glucose homeostasis.

Furthermore, higher HDL has a protective role in neurodegenerative diseases, and exercise has also been shown to have a neuroprotective effect [6]. In this study, we describe a protective association between HDL levels, VO_2_max, and a digit memory test. In addition, we find that younger FitAge Acceleration was associated with better memory test performance, which further supports the beneficial role of physical exercise on cognitive health.

Irisin browning in white adipose tissue increases uncoupling protein 1 levels in white fat cells, and circulating irisin at nanomolar levels enhances energy expenditure, improves glucose tolerance, and exhibits antiobesity and antidiabetic properties [20]. Irisin ablation in mice causes poor browning, hyperlipidemia, insulin resistance, reduced HDL levels, and poor bone strength [21]. We found age-related decreases in irisin levels, which were attenuated by exercise training. We found that circulating blood irisin is closely related to HDL concentration which has previously been reported [21], but the link between irisin to GrimAge Acceleration and FitAge Acceleration is a novel observation. This work further supports the biological importance of irisin to the aging process and aligns with the relationship found between irisin and telomere length [22]. It is possible that our research may provide a rationale for interventions that boost irisin, such as physical exercise, as possible antiaging therapies.

Through this complex investigation, we aim to gain a deeper understanding of how physical fitness is related to DNAm-based aging. We examined the relationship of physical fitness in healthy adults and life-long athletes with different physiological parameters, biochemical tests, memory tests, and DNAm-based biomarkers. The newly created DNAmFitAge outperformed the existing DNA methylation-based biomarkers and revealed that regular exercise is associated with younger biological age, better memory, and more protective blood serum levels. Based on these relationships DNAmFitAge could be an important biological marker of the quality of life.
